# Spatial thinking in infancy: Origins and development of mental rotation between 3 and 10 months of age

**DOI:** 10.1186/s41235-020-00212-x

**Published:** 2020-03-02

**Authors:** Scott P. Johnson, David S. Moore

**Affiliations:** 1 UCLA, Franz Hall, Los Angeles, CA 90095 USA; 2 Pitzer College and Claremont Graduate University, 1050 N. Mills Avenue, Claremont, CA 91711 USA

## Abstract

Mental rotation (MR) is the ability to transform a mental representation of an object so as to accurately predict how the object would look from a different angle (Sci 171:701–703, 1971), and it is involved in a number of important cognitive and behavioral activities. In this review we discuss recent studies that have examined MR in infants and the development of MR across the first year after birth. These studies have produced many conflicting results, yet several tentative conclusions can be reached. First, MR may be operational in infants as young as 3 months of age. Second, there may be sex differences in MR performance in infancy, in general favoring males, as there are in children and in adults. Third, there appear to be multiple influences on infants’ MR performance, including infants’ motor activity, stimulus or task complexity, hormones, and parental attitudes. We conclude by calling for additional research to examine more carefully the causes and consequences of MR abilities early in life.

## Significance Statement

Mental rotation (MR) is a skill we all use, when we are trying to interpret which direction a map indicates we should turn, when we try to determine if an additional piece of luggage will fit into a fixed space in a car, or when we try to imagine how the living room would look with the furniture rearranged. Despite the importance of this ability, its development remains poorly understood. In addition, there is a well-established sex difference on some kinds of MR tasks: on average, adult males outperform adult females. Given the need for MR competence in a number of high-paying occupations—including architecture, surgery, and engineering, for example—continued ignorance about the developmental origins of MR competence stands to perpetuate gender disparities in these fields. A deeper understanding of the emergence of this skill could help reduce MR-related disparities and improve people’s performances in general on tasks that rely on MR processes.

Spatial thinking often involves imagining objects as they might appear from a different viewpoint. *Mental rotation* (MR) refers to the ability to imagine how an object that has been seen from one perspective would look if it were rotated in space into a new orientation and viewed from the new perspective. People use this spatial-cognitive ability in a wide variety of situations, and because of its importance in human activities, MR has been the subject of extensive research. In this article, we review studies of MR in infancy; we identify factors that might influence how MR develops, we discuss links with studies of MR in children and adults, and we discuss some preliminary research that has begun to probe the developmental origins of this form of spatial cognition, in particular the antecedents of individual differences and sex differences in MR.

## Mental Rotation of 3D Objects

Shepard (1978); Shepard & Metzler, 1971) published the first studies to examine adults’ ability to mentally rotate representations of 3-dimensional (3D) objects. Interestingly, the amount of time taken to mentally rotate a representation of a 3D object was found to be a linear function of the angle through which the represented object was rotated. That is, it takes longer to recognize a previously seen object when it has been rotated through, say, a 160-degree angle than when it has been rotated through an 80-degree angle, evidence that MR utilizes analog spatial representations. Studies of MR initially assumed theoretical importance because such findings contradicted views of behaviorists, who insisted that mental representations need not be invoked when trying to explain human behavior. Studies of MR continue to be theoretically important because they shed light on whether cognition might be “analog” and genuinely depictive, or rather should be considered strictly propositional (Pylyshyn, 2002).

Notably, investigations of MR, in particular developmental studies, also have applied and practical importance. MR is disrupted in some developmental disabilities, such as Williams Syndrome (Stinton, Farran, & Courbois, 2008) and dyslexia, because learning to read English and other languages can involve discrimination of mirror-image letters (e.g., b, p, q, and d; Rusiak, Lachmann, Jaskowski, & van Leeuwen, 2007; Rüsseler, Scholz, Jordan, & Quaiser-Pohl, 2005). MR skills are also relevant to science, technology, engineering, and mathematics (STEM) disciplines, so they are likely to be important for various professional careers like architecture, engineering, navigation, and medicine (Kerkman, Wise, & Harwood, 2000; Uttal & Cohen, 2012). MR has been linked to competent performance in geometry (Newcombe, Booth, & Gunderson, 2019) and to mathematical competence more generally (Frick, 2018; Lauer & Lourenco, 2016; van Tetering, van der Donk, de Groot, & Jolles, 2019; Verdine, Golinkoff, Hirsh-Pasek, & Newcombe, 2017; Young, Levine, & Mix, 2018). In addition, training MR has positive effects on children’s math performance (Cheng & Mix, 2014; Cheung, Sung, & Lourenco, 2019). Research on development of spatial-cognitive abilities can have significant impact, therefore, in particular with respect to STEM disciplines. Consistent with this possibility, teenagers who perform better on tasks requiring such abilities are more likely to major in the STEM disciplines in college and to pursue STEM careers (Shea, Lubinski, & Benbow, 2001; Wai, Lubinski, & Benbow, 2009).

An important discovery related to this issue is the finding that there is a relatively large sex difference in performance on MR tasks (Linn & Petersen, 1985; Schöning et al., 2007; Voyer, Voyer, & Bryden, 1995) such that males, on average, outperform females. For example, Kail, Carter, and Pellegrino (1979) reported that nearly one-third of 53 female participants rotated visual stimuli more slowly than the slowest male participant in a group of 51. A meta-analysis of studies on spatial-cognitive ability reported that the most reliable sex differences were found on tasks requiring MR (Voyer et al., 1995). For tasks involving the MR of representations of 3D objects through 3D space, the effect sizes associated with the sex difference are typically large, and larger than the effects of sex on most other types of behavior, including rough-and-tumble play in childhood and aggressive behavior more generally (Collaer & Hines, 1995). The sex difference in MR performance is the largest and one of the most robust of all cognitive sex differences (Linn & Petersen, 1985; Voyer et al., 1995).

MR performance is influenced by both circulating sex hormones (Aleman, Bronk, Kessels, Koppeschaar, & van Honk, 2004; Hampson, 2018; Hausmann, Slabbekoorn, Van Goozen, Cohen-Kettenis, & Güntürkün, 2000) and exposure to sex hormones early in development (Alexander & Son, 2007; Falter, Arroyo, & Davis, 2006; Grimshaw, Sitarenios, & Finegan, 1995). In addition, numerous studies have demonstrated that MR performance can be improved with training (Baenninger & Newcombe, 1989; Cherney, Jagarlamudi, Lawrence, & Shimabuku, 2003; Fernández-Méndez, Contreras, & Elosúa, 2018; Sanz de Acedo Lizarraga & García Ganuza, 2003). These studies provide evidence for both biological and environmental influences on MR, and they raise vital questions about the influences of hormones and experiences on *development* of MR.

Many studies have provided evidence for MR in children between 4 years of age and adolescence (e.g., Estes, 1998; Iachini, Ruggiero, Bartolo, Rapuano, & Ruotolo, 2019; Kail, 1986, 1991; Kail, Pellegrino, & Carter, 1980; Marmor, 1975; Moè, 2016; Titze, Jansen, & Heil, 2010; van Tetering et al., 2019). Other studies failed to find evidence for MR in children younger than 5 years old, and claimed that the failure to find MR in preschoolers reflects a true lack of ability, as opposed to difficulties with the test (e.g., inability to understand instructions; Frick, Ferrara, & Newcombe, 2013; Quaiser-Pohl, Rohe, & Amberger, 2010). Other studies, however, have demonstrated that even 3- and 4-year-olds provide evidence of MR in simplified tasks (Frick, Hansen, & Newcombe, 2013; Krüger, 2018; Krüger, Kaiser, Mahler, Bartels, & Krist, 2014; Levine, Huttenlocher, Taylor, & Langrock, 1999).

Further discussion of MR in preschoolers is beyond the scope of this review, but it is notable that no consistent pattern of sex differences in young children’s MR has been reported. For example, neither Krüger (2018) nor Krüger et al. (2014) observed sex differences in their preschool-aged research participants. However, Levine et al. (1999) reported a substantial advantage for male over female 4.5-year-olds on a spatial transformation task, which included both rotation and translation items, and Frick, Hansen, and Newcombe (2013) reported some sex differences as well with 3-year-old participants. Nonetheless, the sex differences in the latter study were inconsistent, with 3-year-old girls having an advantage in some conditions and 3-year-old boys having an advantage in other conditions. Consequently, the existence of a sex difference in MR competence in children in this age range remains an open question.

## Initial Studies of MR in Infancy

Studies in which infants were presented with rotating objects as visual stimuli generated findings that helped set the stage for research on the development of MR in infancy. For example, using rotating-object stimuli, Kellman (1984) established that 4-month-olds can detect the 3D form of objects rotating around two different axes of rotation (see also Kellman & Short, 1987). Later studies revealed that 2-month-olds presented with kinetic random-dot video displays that specify rotating 3D cubes can perceive the 3D shape of such objects (Arterberry & Yonas, 2000), that 2-month-olds who see video displays of partially occluded 3D shapes rotating around a vertical axis can perceive the unity of the displayed objects despite the presence of the occluders (Johnson, Cohen, Marks, & Johnson, 2003), and that 3- to 5-month-olds seem to recognize objects when multiple views of those objects have been provided (Kraebel & Gerhardstein, 2006; Mash, Arterberry, & Bornstein, 2007), even by rotating the objects around orthogonal axes of rotation (Mash et al., 2007).

Hespos & Rochat, 1997); Rochat and Hespos (1996) demonstrated that infants as young as 4 months can form dynamic mental representations of rotating objects, which they suggested was a “rudimentary” form of MR. In these experiments, infants viewed a two-dimensional (2D) object that underwent rotational motion through a 180-degree arc in the frontal plane. Once the object rotated through approximately 120 degrees of arc, it went behind an occluding screen. After the infants saw the object disappear, the screen was lowered to reveal the object again, half the time in an orientation that an adult observer would expect (if they had tracked the object successfully through its rotational motion), and half the time in an inverted orientation. Infants were reported to look at the inverted object significantly longer than the object in the “expected” orientation, presumably because their expectations about its final orientation had been violated (i.e., a *violation of expectation*, or VoE). The researchers concluded that infants as young as 4 months can *anticipate* the orientation of an object undergoing rotational motion in a 2D plane behind an occluder. Notably, neither of these studies using rotating objects as stimuli reported sex differences in performance.

An experiment with 5-month-old infants reported by Moore and Johnson (2008) differed from these earlier studies in three important ways. First, infants saw video images of 3D stimulus objects rotating in 3D space around a vertical axis. This distinction is potentially important, as the largest effects of sex on older participants’ MR performances have been observed in tasks requiring the rotation of 3D objects through 3D space (Hines, 2013; Levine et al., 1999; Linn & Petersen, 1985; Voyer et al., 1995). Second, Moore and Johnson used a habituation paradigm rather than a VoE paradigm. Habituation paradigms rely on the well-established observation that after repeated exposure to almost any stimulus, infants will exhibit a reduced response to that stimulus, but will continue responding to novel stimuli. Therefore, differential looking times to novel versus familiar stimuli in habituation studies can be ascribed to discrimination and at least some level of recognition (Fantz, 1964). In contrast, the VoE method normally entails an inference that increased looking reflects *expectations*, a prospect that is difficult to confirm independently and that has therefore been criticized by numerous theorists (Bogartz, Shinskey, & Schilling, 2000; Cashon & Cohen, 2000; Charles & Rivera, 2009; Haith, 1998; Kagan, 2019; Moore & Cocas, 2006). Finally, infants in the Moore and Johnson study were required to discriminate between an object and its mirror image, as older participants are required to do in Shepard-style MR studies (Shepard & Cooper, 1982). The Moore and Johnson study thus required infants to discriminate a previously-seen object from its mirror-image, and to recognize that object from a novel perspective.

**Fig. 1:**
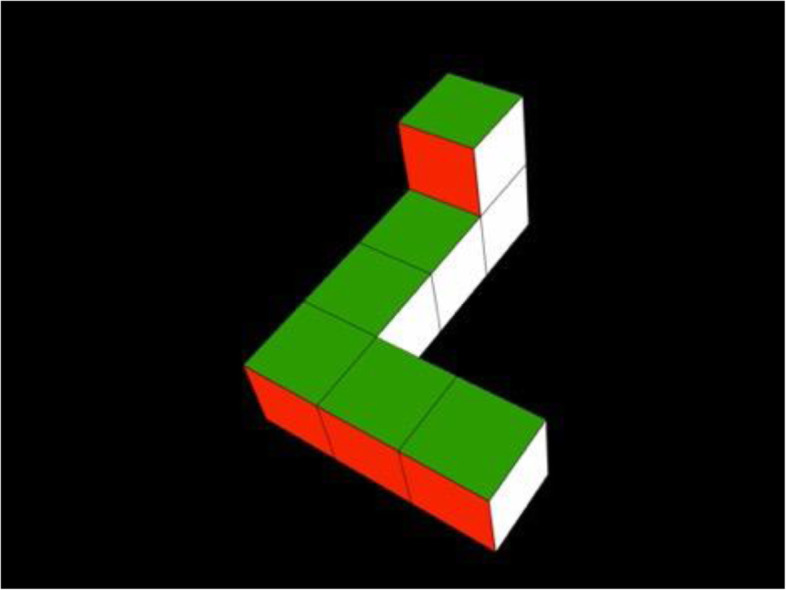
Stimulus object used in Christodoulou, Johnson, Moore, and Moore (2016), Constantinescu, Moore, Johnson, and Hines (2018), Heil, Krüger, Krist, Johnson, and Moore (2018), Moore and Johnson (2008, 2011), and Slone, Moore, and Johnson (2018). Infants were habituated to a video of this object—or its mirror-image—rotating back and forth on its vertical axis through a 240-degree angle. After habituation, infants were tested with a video showing this object (or its mirror-image) rotating back and forth on its vertical axis through the previously unseen 120 degrees of arc. Adapted from Moore and Johnson (2008), Fig. 1

**Fig. 2:**
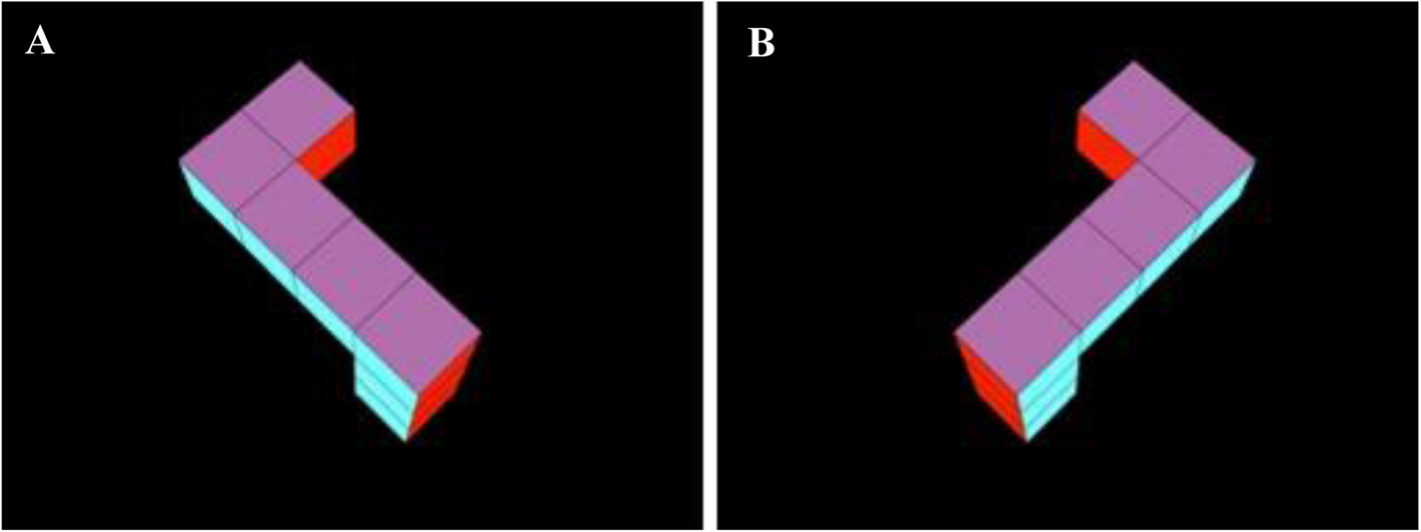
Test stimulus objects used by Christodoulou et al. (2016), Constantinescu et al. (2018), Heil et al. (2018), Moore and Johnson (2008, 2011) and Slone et al. (2018). Habituated infants saw either the object in panel **a** or the object in panel **b** rotating back and forth on its vertical axis through a never-before-seen 120 degrees of arc. Panel **a** represents the “back side” of the habituation object seen in Fig. 1. Panel **b** represents the mirror-image of the object seen in panel **a**. Moore and Johnson (2008, 2011) presented the objects seen in panels **a** and **b** in alternation in a series of 6 test trials. Adapted from Constantinescu et al. (2018), Fig. 2

The infants observed by Moore and Johnson (2008) were initially presented with a series of habituation trials each showing a video representation of a rotating 3D object (see Fig. 1). The object rotated back and forth continuously around the vertical axis through a 240-degree arc. Infants’ looking times at the displays were recorded, and looking times decreased across habituation trials as the infants became habituated to the stimulus. Infants then viewed a series of test trials alternating between two different video displays. In one, they saw the same object they had previously seen in the habituation trials, but now rotating back and forth continuously around the vertical axis through the previously unseen 120 degrees of arc. That is, they saw the familiar object, but now only from the “back side” (see Fig. 2a). (There were no still frames in common between the habituation and test stimuli.) The other test stimulus showed a mirror-image of the familiar object, also rotating through a 120-degree arc (see Fig. 2b). The test objects, therefore, were identical other than in their left-right orientations. Critically, both test stimuli were novel, having never before been seen by any infant. Nonetheless, one of the two test objects was “familiar” in the sense that it was the same object seen during habituation, albeit from a novel perspective. In contrast, the other test object was completely novel, even though it looked and behaved very much like the familiar object (i.e., in the same way that a person’s left and right hands might look very much like one another even though they are easily discriminable).

Moore and Johnson (2008) found that, on average, male 5-month-olds looked significantly longer at the mirror-image test object than at the other test object, whereas female 5-month-olds, on average, looked at both test objects for about the same amount of time. Likewise, 70% of the male babies spent more time fixating the mirror-image test object whereas only 45% of the female babies did. The female and male infants took about the same amount of time to habituate to the original object. Moore and Johnson concluded that the male infants’ preferences for the mirror-image test object indicated that they were relatively uninterested in the habituation object when it was seen in the test trials, even though they had never seen that object from the novel test-trial perspective. That is, they seemed to recognize the habituation object when it was seen from this novel perspective. Presumably, this requires MR of a representation of the habituation object, so as to allow a comparison of that representation with the visible test object, or MR of a representation of the test object, so as to allow a comparison with a representation of the habituation object, or both.

**Fig. 3:**
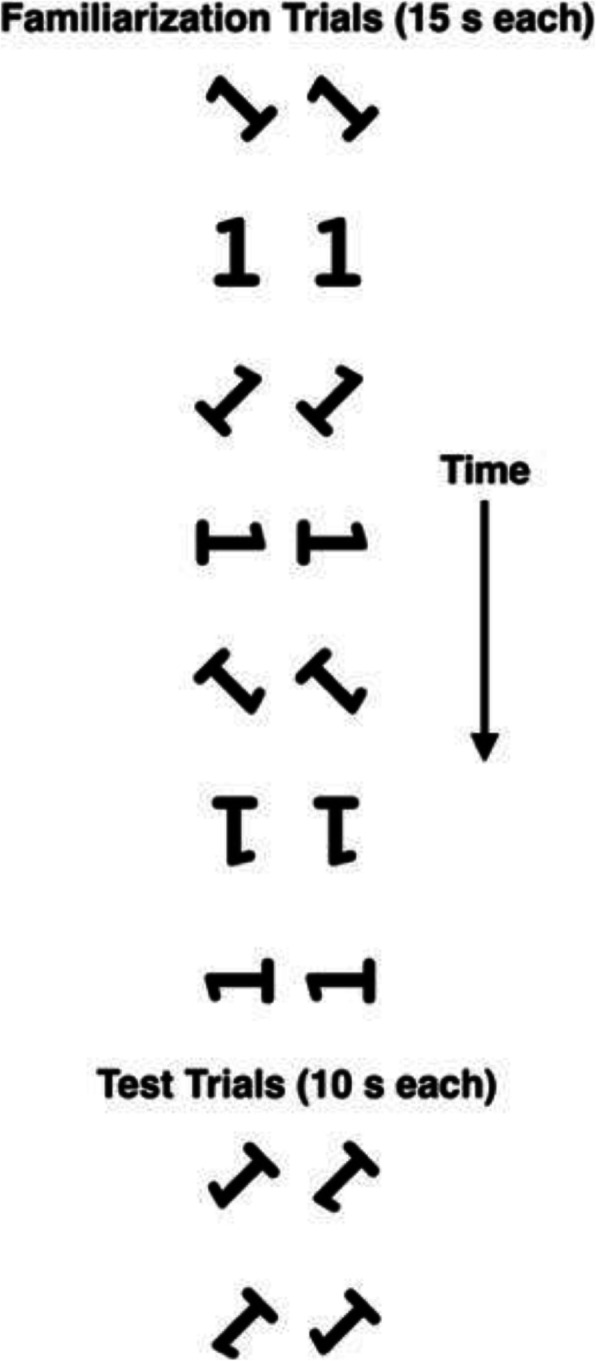
Schematic depiction of Quinn and Liben’s (2008) experimental design. Infants were presented with seven different rotations of the number 1 stimulus (or its mirror image) during familiarization, with two identical copies of each stimulus presented on each trial. For familiarization, [Quinn & Liben] randomly selected seven of the eight possible rotations and their order of presentation for each infant in the female group and a corresponding infant in the male group. The test stimuli paired the novel rotation of the familiar stimulus with its mirror image” (Quinn & Liben, p. 1068). Adapted from Quinn and Liben (2008), Fig. 2

By coincidence, a second paper on infants’ MR was published alongside the Moore and Johnson (2008) report in the same issue of *Psychological Science*. Quinn and Liben (2008) tested 3- to 4-month-olds with stimuli consisting of pairs of identical static images of a 2D object presented over multiple familiarization trials, in which each stimulus pair was presented in a new orientation, as if the objects had been rotated around a clock face (see Fig. 3). These familiarization trials were followed by test trials that presented an image of the object (and its mirror-image) rotated through 2D space into yet another novel position. Quinn and Liben found that female infants had no visual preference for a novel view of a previously-seen object or a mirror-image of that object, but male infants spent significantly more time looking at the mirror-image of the object. In addition, whereas 11 out of 12 male infants looked longer at the mirror-image object, only 5 out of 12 female infants did. Quinn and Liben (2014) subsequently demonstrated a male advantage in two older age groups as well, 6- to 7-month-olds and 9- to 10-month-olds.

## Further Evidence on the Emergence of MR in Infancy

Studies of MR in infants since the Moore and Johnson (2008) and Quinn and Liben (2008) experiments have addressed a number of important questions, including the replicability of the effect in general and the sex difference in particular, as well as antecedent factors that might contribute to the development of this important skill.

Moore and Johnson (2011) tested 3-month-olds using the methods and stimuli from the Moore and Johnson (2008) study, and again observed a sex difference. Female infants looked at the two test stimuli for about the same amount of time, but the 3-month-old male infants looked longer at the habituation object in the new orientation than they did at the mirror-image object. Moore and Johnson (2011) interpreted this result as meaning the MR task was more difficult for the younger infants than for the older infants. Hunter, Ames, and Koopman (1983) successfully explained some variation in infants’ looking times by positing that their fixation durations are affected by factors such as familiarization time, stimulus complexity, and the infants’ ages. More specifically, they argued that familiarity preferences are more likely than novelty preferences when infants have not finished processing a stimulus. Consequently, if a stimulus is complex, if an infant is young (and therefore less able to process information quickly), or if an infant is exposed to a stimulus for a relatively short period of time, that infant will be more likely to fixate a familiar, but incompletely processed, stimulus than a novel stimulus (Hunter & Ames, 1988). Consistent with this possibility, Colombo (1995) noted that infants look longer when slower processing speeds lead them to require more time to look at and process information about stimulus properties. Because the 3-month-old males in the Moore and Johnson (2011) study demonstrated a statistically reliable preference for the familiar test stimuli, they—like the 5-month-olds in the Moore and Johnson (2008) study—did not treat the test stimuli equally, as they would have done if they failed to recognize the habituation object. Thus, we again concluded that male 3-month-olds, but not female 3-month-olds, were capable of MR, even if their familiarity preference suggested that the task was more difficult for them than it was for the older infants tested in 2008.

The 3-month-old males’ familiarity preference in the Moore and Johnson (2011) study also suggests that the task is more difficult than the Quinn and Liben (2008) task, in which similarly-aged infants showed a novelty preference. This effect could perhaps reflect the fact that while the Moore and Johnson task requires recognition of a 3D object rotated though 3D space, the Quinn and Liben task requires recognition of a 2D object rotated through 2D space. Moreover, a study that utilized a two-monitor display—a methodological feature that might arguably have been expected to make the task more difficult—revealed a significant familiarity preference among 5-month-olds (Christodoulou et al., 2016). Likewise, a study of 5-month-olds conducted in Germany found a significant familiarity preference among infants tested using the same paradigm, even among female infants (Erdmann, Kavšek, & Heil, 2018).

Other researchers have used different methods to study babies 6 months of age or older. Using a VoE paradigm, Möhring and Frick (2013) found evidence of MR in 6-month-olds, but only in 6-month-olds who had previously been given an opportunity to manually explore an object before being tested with it. Likewise, Lauer, Udelson, Jeon, and Lourenco (2015) reported that infants as young as 6 months can form mental representations of the orientation of a 2D object and use those mental representations to discriminate the object from its mirror image, a finding these researchers considered convergent evidence for MR competence in infancy.

Experiments with older infants have explored the possibility that MR competence in the first year after birth is related to gross motor development. In a study with 9-month-olds, Schwarzer, Freitag, Buckel, and Lofruthe (2013) found that MR performance was related to crawling ability, such that only infants who had started to crawl had a significant preference for the novel, mirror-image test object. Schwarzer, Freitag, and Schum (2013) confirmed this finding while also discovering that among non-crawling 9-month-olds, only those who spontaneously explored a collection of toy blocks with their hands showed a significant preference for the novel, mirror-image test object. In another follow-up study, Gerhard and Schwarzer (2018) found that while non-crawling 9-month-olds spent approximately equal amounts of time looking at novel views of familiar and mirror-image test objects, 9-month-olds with crawling experience spent significantly more time looking at the mirror-image object in a condition requiring a small degree of MR, but significantly more time looking at the *familiar* object (seen from a new perspective) in a condition requiring a larger degree of MR. Additional results consistent with these findings were reported by Frick and Möhring (2013), who found that among 10-month-olds, MR performance on a VoE task was related to extent of motor development reported by parents on a questionnaire. Taken together, this collection of results suggests that at least some infants become capable of MR in the first year after birth.

## Sex Differences in MR in Infants

As noted earlier, numerous studies of MR in adults have revealed sex differences favoring males, and meta-analyses of studies in this domain have confirmed that when participants are asked to rotate mental representations of 3D objects through 3D space, the magnitude of this sex difference is large and the effect is robust (Linn & Petersen, 1985; Voyer et al., 1995). In contrast, studies of young children have provided less consistent results. Following their 1995 meta-analysis, Voyer and colleagues concluded that a sex difference in MR does not appear prior to about 10 years of age, and more recent research by Krüger (2018); Krüger et al., 2014) likewise found no sex differences in MR in a population of preschoolers. Some researchers (Frick, Hansen, & Newcombe, 2013; Levine et al., 1999) have reported sex differences in children as young as 3 or 4.5 years of age, but these were inconsistent in direction and across conditions.

Several studies have found sex differences in infants younger than 12 months but others have not. Early work on rotating objects did not reveal sex differences (Hespos & Rochat, 1997; Mash et al., 2007; Rochat & Hespos, 1996), but both of the first studies of MR that required infants to recognize a rotated object and discriminate it from its mirror image reported a sex difference favoring males (Moore & Johnson, 2008; Quinn & Liben, 2008). Since then, three additional studies in our labs (Constantinescu et al., 2018; Moore & Johnson, 2011; Moore, Johnson, & Moore, 2020) and three additional studies in three other labs (Kaaz & Heil, 2019; Lauer et al., 2015; Quinn & Liben, 2014) have reported a male advantage in MR in infants 10 months of age or younger. In contrast to these eight studies, two studies in our labs (Christodoulou et al., 2016; Slone et al., 2018) and six studies in three other labs (Erdmann et al., 2018; Frick & Möhring, 2013; Gerhard & Schwarzer, 2018; Möhring & Frick, 2013; Schwarzer et al., 2013, b) have reported no sex differences in infants from this age range. Given this relatively even distribution of findings, we agree with the conclusion offered in Lauer and colleagues’ recent meta-analytic review on the development of gender differences in spatial reasoning: “further investigation of infants’ mental rotation abilities will be necessary to determine whether gender differences in implicit mental rotation performance are indeed present in the first year of life and, if so, whether these gender differences represent the origins of the later male advantage in explicit mental rotation performance” (Lauer, Yhang, & Lourenco, 2019, p. 550; see also Levine, Foley, Lourenco, Ehrlich, & Ratliff, 2016).

Despite the fact that it is too early to say with confidence whether sex differences in MR competence are present in the first year after birth, there are some observations worth noting at this juncture. First, although several studies have failed to find evidence of a sex difference in MR competence, those that have found a sex difference have consistently found an advantage for male infants. In seven out of eight of these cases, male infants on average have responded in significantly different ways to familiar versus mirror-image objects, whereas female infants on average have consistently treated these objects similarly. Although the data from the eighth study (Lauer et al., 2015) indicated that both male and female infants discriminated non-mirror from mirror-image objects, a main effect of sex still indicated that boys spent significantly more time than girls looking at displays containing mirror-image objects. Thus, in the studies that have detected sex differences to date, all eight have revealed effects in the same direction, in favor of males.

Second, it is important to keep in mind that the absence of evidence (in 7 out of 8 studies) that female infants discriminate mirror- from nonmirror-images cannot be taken as confirmation that these infants are *not* capable of MR. As Levine et al. (2016) noted, “There are many reasons why [female] infants may not look longer at the novel mirror image stimulus … they may find both test stimuli interesting—after all, both are presented [from a perspective] that was not seen during the habituation trials. … This possibility would be consistent with a sex difference, but not one that reflects an ability of male but not female infants to mentally rotate figures” (2016, p. 5–6). The Lauer et al. (2015) finding that female infants preferred displays containing mirror-images over displays containing only nonmirror-images (though significantly less than did male infants) is consistent with the possibility that female infants are capable of MR, even if they do not consistently provide evidence of that competence.

Finally, it is worth considering that it is never the case that all male infants outperform all female infants in these sorts of tasks. As the behavioral neuroendocrinologist Elizabeth Hampson has pointed out, the kinds of differences described in this section might “reflect the operation of graded factors that covary with sex (e.g. ambient hormone concentrations), not sex as a categorical variable. Indeed, sex is frequently only an imperfect proxy for factors such as hormones that explain between-sex and within-sex variation better than binary ‘sex’ alone” (Hampson, 2018, p. 49).

## Factors Affecting Infants’ MR Performances

In this section we consider studies that have examined factors related to infants’ MR and its development, in particular the means by which experience (i.e., motor activity) and stimulus or task complexity influence infants’ ability to mentally rotate complex objects in 3D space. In addition, there is evidence that hormones and socialization modulate MR in 5-month-olds. We will consider each of these factors in turn.

Notably, we lack a coherent account of how these different factors, alone or in tandem, operate to affect MR and its development, either in individuals or across groups of male and female infants. For example, hormones affect development in distinct ways for females and males (Hines, 2013, 2015) because specific hormones have targeted developmental effects, and because hormone levels are different for the two genders. In contrast, it is not clear how motor activity would affect MR performance differently for infant females and males.

### Motor Activity

The hypothesis that motor activity would influence performance on perceptual/cognitive tasks can be traced to Piaget (1952; Piaget & Inhelder, 1956), and research on MR in children and adults revealed that MR involving representations of hands is influenced by the posture of participants’ own hands (Funk, Brugger, & Wilkening, 2005). Similarly, young children’s MR of representations of objects is influenced by their concurrent manual activity (Frick, Daum, Walser, & Mast, 2009; Frick, Daum, Wilson, & Wilkening, 2009). In addition, as described previously, Schwarzer, Freitag, Buckel, and Lofruthe (2013) examined the relation between crawling experience and MR competence in 9-month-olds, and found that infants who had begun crawling spent more time looking at a mirror-image test object than at novel views of a habituation object, thereby providing evidence of MR; in contrast, infants of the same age who had not yet had experience crawling treated the test stimuli identically. Schwarzer and colleagues subsequently replicated and extended this effect (Gerhard & Schwarzer, 2018; Schwarzer, Freitag, & Schum, 2013).

Relatedly, Soska, Adolph, and Johnson (2010) reported that 4.5- to 7.5-month-olds’ 3D object completion (i.e., perception of objects as coherent in 3D space from a limited viewpoint) was aided by experience with visually coordinated manual object exploration, which suggested to Schwarzer, Freitag, and Schum (2013) that this kind of manual experience might facilitate MR performance in infants as well. Likewise, Möhring and Frick (2013) hypothesized that manual experience with an object would influence 6-month-olds’ subsequent MR of that object (cf. Frick & Wang, 2014). Using a VoE method, these researchers discovered that infants given hands-on experience with an object prior to an MR test spent more time looking at a mirror-image test object than at a novel view of the previously-seen object, while infants who merely *saw* the object prior to the testing sequence failed to discriminate the test objects. Schwarzer, Freitag, and Schum (2013) study of the effects of manual object exploration on MR revealed a similar effect in non-crawling 9-month-olds, and a follow-up study by Frick and Möhring (2013) found a positive relation between parent-reported motor development in infants and their performance in the VoE MR task. Taken together, these results strongly suggest that gross and fine motor experiences both influence MR competence in infancy.

**Fig. 4:**
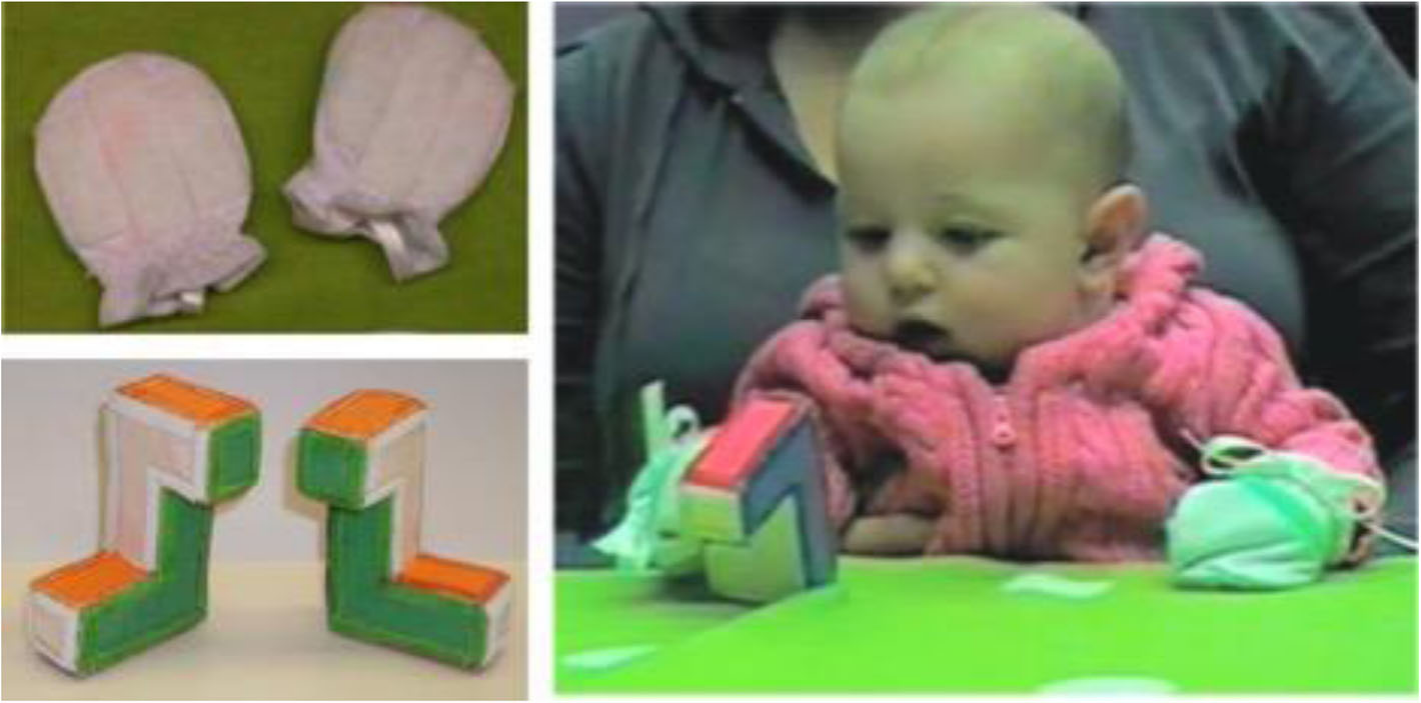
Photographs illustrating Slone et al.’s (2018) object exploration task. Top left: Velcro mittens worn by infants in the task. Bottom left: Objects given to the infants during the task. Right: An infant engaged in the object exploration task. (The parent of this infant provided written informed consent to publish this image). Reproduced from Slone et al. (2018), Fig. 2

A study of younger infants is consistent with this possibility. Slone et al. (2018) used a “sticky mittens” procedure (Needham, Barrett, & Peterman, 2002) to give 4-month-old infants manual experience with objects prior to when they would ordinarily develop the ability to manually explore objects spontaneously. By affixing “loop” and “hook” Velcro strips to cloth mittens and small objects, respectively, and then fitting infant participants with the mittens, 4-month-olds were enabled to “pick up” objects simply by making contact with them, even absent the manual dexterity that allows older infants to actually grasp objects (see Fig. 4). In this case, infants interacted with objects that closely resembled the habituation and test objects described previously for studies of infant MR (Figs. 1 and 2). The objects’ configurations were identical, as were the patterns of coloring on the objects’ different faces. Following this experience, infants in an experimental group were tested with the method described by Moore and Johnson (2008). There was a statistically significant relation between spontaneous object engagement (defined as coordinated looking and touching plus looking alone plus touching alone) and novelty preference at test; infants who had “sticky mittens” experiences prior to being tested and who exhibited more engagement with the object had stronger preferences for the novel (mirror-image) object. This effect may have stemmed directly from differences in visual-manual experiences, general attention or activity level, or some combination thereof (though there is little evidence that sticky mittens “training” facilitated MR performance). Thus, available evidence suggests that motor developments—and more generally, the visual, proprioceptive, and multimodal experiences they provide (Bahrick & Lickliter, 2014)—are important contributors to the development of MR competence.

### Stimulus or Task Complexity

Another factor that appears to influence MR competence is stimulus or task complexity. Complexity must be understood in this context as varying as a function of an infants’ developmental state; a stimulus or task that is complex from one infant’s perspective might be simple from the perspective of an older infant. Accordingly, the same stimulus or task that yielded novelty preferences (on average) from 5-month-old males in the Moore and Johnson (2008) study yielded familiarity preferences (on average) from 3-month-old males in the Moore and Johnson (2011) study. Consistent with the Hunter and Ames model (Hunter et al., 1983; Hunter & Ames, 1988), younger infants’ preferences for the familiar, previously-seen test object suggests that they had not completed processing the stimulus during the initial phases of the experiment, presumably because it was a relatively complex stimulus from their perspective. Thus, the Moore and Johnson task appears to be more difficult for younger infants.

Gerhard and Schwarzer (2018) provided another example of how variations in difficulty can influence the emergence of familiarity versus novelty preferences. These researchers reported that among 9-month-olds with crawling experience, a task requiring MR through a minimal angle of rotation generated the expected novelty preference at test, but a task requiring MR through a much larger angle (54°) generated a *familiarity* preference at test. Given the well-established finding that for adults, MR of an object through progressively larger angles takes progressively more time—and that these are therefore arguably more difficult tasks—the finding that a task requiring a larger angle of rotation yielded familiarity preferences in 9-month-olds supports the claim that familiarity preferences are indicative of increased task difficulty. Moreover, in an earlier study, Schwarzer, Freitag, and Schum (2013) found that 9-month-olds with significant motor experience (i.e., crawling infants, or non-crawling infants who displayed relatively high levels of spontaneous manual object exploration) exhibited a novelty preference, but non-crawling 9-month-olds who did not spontaneously explore objects with their hands exhibited a familiarity preference; this result, too, is consistent with the idea that a given task is more complex for an infant at an earlier developmental level.

Although there have been varying reasons why different experimental tasks might have been more challenging for different groups of infants across the tasks in these investigations, in each study about which we have sufficient information to judge,^1^ more complex tasks (given the participants’ developmental states) have always been more likely to yield familiarity preferences. Consequently, it seems reasonable to conclude that stimulus or task complexity influences MR competence in infants much as it does in older populations (e.g., Bethell-Fox & Shepard, 1988).

### Hormones

Relatively large sex differences have consistently been observed in such characteristics as height, sexual orientation, and gender identity, and the development of these characteristics appears to be influenced by exposure to testosterone early in life (Hines, 2015). Consequently, it is possible that a sex difference in MR, too, is affected by hormones. In adult women, normal hormonal variations across the menstrual cycle are correlated with performances on MR tasks (Hausmann et al., 2000), and two double-blind placebo-controlled experiments have demonstrated that a single half-milligram dose of testosterone can temporarily improve healthy young women’s performances on an MR task (Aleman et al., 2004; Pintzka, Evensmoen, Lehn, & Håberg, 2016). Thus, testosterone appears to influence spatial ability via an *activating* role in the central nervous system. In addition, variation in exposure to testosterone may contribute to later-emerging sex differences by influencing variation in the *organization* of the developing nervous system early in life. Children’s gender-related playmate and toy preferences, for example, are affected by prenatal testosterone exposure (Constantinescu & Hines, 2012).

In contrast to these relatively clear organizational effects, the influence of prenatal and early postnatal hormones on later-appearing *cognitive* sex differences is less clear (Hines, 2010, 2015). Hormones such as testosterone do many different things early in life—including “altering cell numbers in specific brain structures, inducing outgrowth of axons and dendrites, supporting synaptogenesis, regulating cell death, and affecting axonal guidance” (Moore, 2012, p. 415)—and they can produce different effects in different cell types (Li & Al-Azzawi, 2009). Notwithstanding this lack of specificity-of-action, prenatal androgen exposure was correlated with speed of MR in one study of 7-year-old girls (Grimshaw et al., 1995) and some studies (Berenbaum, Korman Bryk, & Beltz, 2012; Hampson, Rovet, & Altmann, 1998; Resnick, Berenbaum, Gottesman, & Bouchard, 1986) have found that prenatal exposure to abnormally high levels of testosterone in females with congenital adrenal hyperplasia (leading to an excess of testosterone) is associated with better MR performances (but see Helleday, Bartfai, Ritzen, & Forsman, 1994; Hines et al., 2003; and Malouf, Migeon, Carson, Pertrucci, & Wisniewski, 2006 for contradictory findings). Early-life exposure to hormones, therefore, may be related to infants’ MR performance. Notably, prenatal and early postnatal periods in human development are characterized by dramatically different concentrations of gonadal steroid hormones in male versus female fetuses/newborns (Corbier, Edwards, & Roffi, 1992; Gendrel, Chaussain, Roger, & Job, 1980; Hammond, Koivisto, Kouvalainen, & Vihko, 1979; Lamminmäki et al., 2012; Reyes, Boroditsky, Winter, & Faiman, 1974).

A recent study examined the relation between levels of testosterone and estradiol measured in amniotic fluid surrounding 14- to 15-week-old fetuses, and the MR performances of these individuals approximately 1 year later, when they were 6-month-old babies (Erdmann et al., 2019). Although this study did not find any sex differences in behavior, MR performances of boys were nonetheless correlated with their exposure to testosterone (but not estradiol) *in utero*. In contrast, MR performances of girls were correlated with prenatal estradiol (but not testosterone) exposure.

Another recent study examined the relation between MR performances in 5- to 6-month-olds and their levels of salivary testosterone measured several months earlier, when they were 1 to 2.5 months of age (Constantinescu et al., 2018). This period between the first and third postnatal months has been called “mini-puberty” (Lamminmäki et al., 2012) because of a surge in testosterone that is especially large in boys at this time (Corbier et al., 1992; Gendrel et al., 1980; Hammond et al., 1979). The timing of this surge is thought to be potentially important to human cognitive development (Lyall et al., 2015) because it occurs during a period of rapid cortical development, some of which is occurring in regions of the brain that appear to be active during MR in adults (Gogos et al., 2010; Schendan & Stern, 2007; Schöning et al., 2007). Constantinescu et al. (2018) replicated the sex difference in MR performance reported by Moore and Johnson (2008), and they also found a significant positive correlation between boys’ early postnatal testosterone exposure and their MR performance when tested at 5 months. Thus, hormonal events during “mini-puberty” might have lasting organizational influences on boys’ central nervous systems, influences that affect their MR competence later in infancy. The specific mechanisms by which hormones might influence later spatial cognition remain unknown at present, but a candidate mechanism could involve hormonal modulation of gene transcription in neurons in particular brain regions (Hampson, 2018; Hara, Waters, McEwen, & Morrison, 2015).

### Parental Attitudes

Constantinescu et al. (2018) also examined how factors related to socialization might modulate infants’ MR performances. To this end, they provided parents of tested infants with the Child Gender Socialization Scale (the CGS Scale; Blakemore & Hill, 2008), designed to assess the extent to which parents’ attitudes are gender-stereotypical. This scale consists of 28 items that have been demonstrated to differentiate between boys’ parents and girls’ parents, and between parents with more versus less traditional ideas about gendered activities such as taking ballet lessons or playing with toy cars. Interestingly, there was a significant correlation between 5-month-old girls’ MR performance and their parents’ scores on the “Disapproval of other-gender characteristics” subscale of the CGS Scale. Specifically, parents with less traditional ideas about gendered activities—that is, parents who were more likely to say they would approve of a daughter exhibiting male-typical behaviors like playing football or playing with toy guns—were more likely to have 5-month-old daughters who provided evidence of successful MR in our standard task. The correlation between parental attitudes and MR performance was present in girls only, for reasons that remain unclear at the moment.

Although we do not currently know how parental attitudes could influence infants’ MR performances, it seems reasonable to expect that multiple social and other experiential factors contribute to the development of MR competence, and to the development of the sex difference in this skill that emerges later in life (Halpern, 2000; Lauer et al., 2019; Levine et al., 2016). Experiences with particular stimuli and tasks are known to influence children’s and adults’ performances on spatial ability tests in general (Baenninger & Newcombe, 1995), and individuals who choose to participate in activities that require spatial skills have better MR abilities (Peters, Lehmann, Takahira, Takeuchi, & Jordan, 2006; Quaiser-Pohl & Lehmann, 2002; Voyer, Nolan, & Voyer, 2000). For example, experience with computers has been shown to mediate the sex difference in MR ability (Terlecki & Newcombe, 2005). Furthermore, experimental protocols designed to *train* spatial-cognitive skills improve both males’ and females’ performances on spatial tasks (Baenninger & Newcombe, 1989; Sanz de Acedo Lizarraga & García Ganuza, 2003).

Male and female individuals encounter different social worlds, even in early infancy (Donovan, Taylor, & Leavitt, 2007; Stern & Karraker, 1989). It is likely, therefore, that any sex difference in MR competence reflects the effects of these differing experiences as well as the effects of the stereotype-based expectations to which individuals are exposed (Levine et al., 2016). Women have been shown to underperform on MR tasks when they are provided with a reminder about their gender prior to being tested (McGlone & Aronson, 2006). Similarly, when women are explicitly told that “men outperform women” on a difficult visuospatial task (Campbell & Collaer, 2009) or that “men are better” on an MR task (Heil, Jansen, Quaiser-Pohl, & Neuburger, 2012), their performances are negatively affected, and in childhood, anxiety about spatial ability appears to impair MR performance in girls, but not in boys (Ramirez, Gunderson, Levine, & Beilock, 2012). Thus, experiences—including the beliefs we have about ourselves and that others have about us—can be expected to contribute to the development of MR competence, as well as to spatial-cognitive competence more generally.

## Conclusion

Although the data collected to date suggest that MR can be detected as early as 3 months of age, we remain largely ignorant about the mechanisms by which this ability develops. Clearly, important developmental events are occurring either prenatally or in the first 3 months of postnatal life. Further research on the role of genetic, hormonal, and experiential factors in the development of MR competence will be required to illuminate these developmental processes. In addition, it remains unclear how MR performance in infancy may vary depending on the size of the angle through which MR is required. One study found that 5-month-old males were able to recognize an object that had been rotated through a 30° angle of rotation (Moore et al., 2020), and older infants were able to recognize an object across a much larger rotational gap (54°) (Gerhard & Schwarzer, 2018), suggesting that MR in infancy operates across such gaps. Specific effects of varying angular disparities, however, remain unknown. Likewise, we remain unsure if the gender difference in MR that is detectable in older populations is present in infants, and if so, what the underlying causes of this difference might be. Most gender differences in human behavior result from numerous factors interacting over time (Moore, 2012), and the factors that contribute to differences in MR competence are likely those that contribute to gender difference more broadly, including early exposure to steroids like testosterone and socialization by parents, siblings, and teachers, as well as self-socialization based on an individual’s understanding of gender (Halpern, 2000; Hines, 2015). The interactions that drive development of MR competence early in life are likely to be complex, but research that elucidates these processes can be expected to have significant payoffs, because understanding the development of this important skill will facilitate the creation of interventions that can improve performances and open doors to productive careers.

## Data Availability

N/A.

## Footnotes

1 Erdmann et al. (2018) reported an unexpected familiarity preference in 5-month-olds exposed to the Moore and Johnson (2008) stimuli, but their method differed from the method used in other laboratories (e.g., Constantinescu et al., 2018; Slone et al., 2018) in both known and unknown ways, hindering our ability to interpret this finding.
